# A Comparative Study of Topical Insulin Application Over Diabetic Foot Ulcers Versus a Normal Saline Dressing

**DOI:** 10.7759/cureus.105941

**Published:** 2026-03-26

**Authors:** Alljin Sanjoe P, Manjunath S Kotennavar, Aravind V Patil, Sanjeev Rathod, Pradeep P Jaju, Manjunath S Savant, Veena Ghanteppagol, Smit S Parikh, Nilan Sabbaiah

**Affiliations:** 1 General Surgery, Shri BM Patil Medical College, Hospital and Research Centre, Bijapur Lingayat District Educational Association (BLDE) (Deemed to be University), Vijayapura, IND

**Keywords:** diabetic foot ulcer, granulation tissue, topical insulin, ulcer dressing, wound healing

## Abstract

Introduction: Diabetic foot ulcer (DFU) is a major complication of diabetes mellitus and an important cause of prolonged hospitalization and lower limb amputation. Delayed wound healing in diabetic ulcers is associated with impaired angiogenesis, chronic inflammation, and reduced cellular proliferation. Topical insulin has been reported to promote fibroblast proliferation, collagen synthesis, and angiogenesis, thereby enhancing wound healing. However, clinical evidence remains limited and heterogeneous in terms of study design and outcomes. The present study aimed to compare the effectiveness of a topical insulin dressing with a conventional normal saline dressing in patients with DFUs.

Materials and methods: A non-randomized prospective comparative study was conducted in the Department of General Surgery at Shri B.M. Patil Medical College Hospital and Research Centre, Vijayapura. A total of 94 consecutive patients with Wagner grade 1 and 2 DFUs were included. Patients in the insulin dressing group received topical insulin dressings, while the comparison group received conventional normal saline dressings. Allocation to treatment groups was based on the treating surgeon's preference as part of routine clinical practice (non-randomized). Wound assessments were performed weekly to evaluate granulation tissue formation, wound surface area, and the presence of wound discharge. Additionally, percentage wound reduction, duration of hospital stay, time to complete ulcer closure, and final surgical outcomes were assessed.

Results: Baseline characteristics were comparable between the groups. The insulin group demonstrated significantly higher granulation tissue formation from week 1 onward compared with the saline group. Mean wound reduction was significantly greater in the insulin group (48.44 ± 5.76%) compared with the saline group (19.15 ± 4.34%). Persistent wound discharge was observed in 11 (23.4%) patients in the saline group at week 1 and 7 (14.9%) at week 2, while no patients in the insulin group had discharge during these periods. The mean hospital stay was shorter in the insulin group (14.89 ± 1.90 days) compared with the saline group (19.21 ± 2.17 days). Similarly, time to complete ulcer closure was shorter in the insulin group (33.4 ± 3.7 days) than in the saline group (52.8 ± 5.2 days). No hypoglycaemia or systemic adverse effects were observed.

Conclusion: Topical insulin dressing was associated with improved wound healing outcomes in patients with DFUs and appears to be a safe adjunct to conventional wound care. However, given the non-randomized design, these findings should be interpreted as associative rather than causal, and further randomized controlled studies are required.

## Introduction

Diabetes mellitus is one of the most important global health challenges of the 21st century, affecting both developed and developing countries [1]. According to the International Diabetes Federation, approximately 537 million adults were living with diabetes in 2021, and this number is projected to rise to nearly 783 million by 2045 [2]. Chronic hyperglycaemia resulting from defects in insulin secretion, insulin action, or both leads to several long-term complications, among which diabetic foot ulcer (DFU) is one of the most serious and disabling [3]. Epidemiological studies indicate that diabetic patients have a lifetime risk of foot ulcer development of nearly 15-25% [4]. Peripheral neuropathy and peripheral vascular disease predispose patients to repeated trauma, impaired tissue perfusion, and delayed wound healing, ultimately leading to chronic ulcers and infection [5]. DFUs precede the majority of diabetes-related lower limb amputations and are associated with prolonged hospitalization, high treatment costs, and reduced quality of life [6].

The pathophysiology of DFUs is complex and involves metabolic, vascular, and immunological disturbances that disrupt the normal wound healing cascade [3]. Persistent hyperglycaemia results in the accumulation of advanced glycation end products, oxidative stress, and microvascular dysfunction, all of which impair tissue repair mechanisms [7]. In addition, dysregulation of the inflammatory phase leads to sustained elevation of pro-inflammatory cytokines that delay healing [8]. Reduced fibroblast proliferation, impaired collagen synthesis, decreased angiogenesis due to reduced vascular endothelial growth factor activity, and increased matrix metalloproteinase activity further compromise tissue regeneration [9]. Because of these alterations, many DFUs heal slowly even with conventional treatment, which typically includes wound debridement, infection control, pressure off-loading, glycaemic optimization, and saline dressings [10].

In recent years, insulin has been recognized to possess several biological effects beyond glycaemic regulation that may enhance wound healing [11]. Studies have demonstrated that insulin promotes keratinocyte migration, fibroblast proliferation, collagen synthesis, and angiogenesis, while also modulating the inflammatory response [12,13]. These actions are mediated through activation of intracellular signalling pathways such as PI3K-Akt and MAPK-ERK in cells involved in tissue repair [14]. The presence of insulin receptors on keratinocytes, fibroblasts, and endothelial cells provides a biological basis for the topical application of insulin directly at the wound site [13]. Topical insulin is thought to enhance granulation tissue formation and tissue regeneration while minimizing systemic absorption and the risk of hypoglycaemia [11,15].

However, existing clinical evidence on topical insulin remains limited by small sample sizes, heterogeneity in study design, and variability in outcome assessment. Furthermore, many studies lack methodological rigor, including standardized protocols and control of confounding variables. Therefore, the present study aimed to compare topical insulin dressing with conventional normal saline dressing in patients with Wagner grade 1 and 2 DFUs, with clearly defined primary and secondary outcomes, including granulation tissue formation, wound discharge resolution, wound size reduction, duration of hospital stay, time to complete ulcer closure, and final surgical outcomes.

This research work was originally conducted as part of a postgraduate dissertation submitted to the Department of General Surgery, BLDE (Deemed to be University), Vijayapura, Karnataka, India.

## Materials and methods

This non-randomized prospective comparative study was conducted in the Department of General Surgery at Bijapur Lingayat District Educational Association (BLDE) (Deemed to be University), Shri B.M. Patil Medical College Hospital and Research Centre, Vijayapura, over a period of 18 months from June 2024 to December 2025. Ethical approval was obtained from the Institutional Ethics Committee (IEC-SBMPMC/098/2023-24), and written informed consent was obtained from all participants prior to enrolment.

Sample size was calculated using the formula for comparison of two independent means:

$n = \frac{2 (Z_{\alpha/2} + Z_{\beta})^2 \sigma^2}{\Delta^2}$

where Zα/2​=1.96 for a 95% confidence level, Zβ​ = 0.84 for 80% statistical power, σ represents the pooled standard deviation, and Δ represents the expected difference between the two groups.

The calculation was based on the findings reported by Biradar D et al., which showed a mean ulcer surface area at day 7 of 2.06 ± 1.43 cm² in the insulin group and 2.98 ± 1.78 cm² in the control group [15]. Using these values, the pooled standard deviation was calculated as 1.61, and the expected difference between groups was 0.92 cm². Assuming a 95% confidence level and 80% statistical power, the calculated sample size was approximately 48 patients per group. This was rounded to 47 patients in each group, giving a total sample size of 94 patients.

Patients with Wagner grades 1 and 2 DFUs were included in the study [16]. Patients with a known allergy to insulin, skin malignancy at the ulcer site, or severe peripheral vascular disease defined by an ankle-brachial pressure index (ABPI) less than 0.5 were excluded from the study [17].

Patients were enrolled consecutively after applying the eligibility criteria. Patients in the insulin dressing group received topical insulin dressings, whereas those in the conventional group received normal saline dressings. Allocation to treatment groups was based on the treating surgeon’s routine clinical preference, and no randomization was performed.

At baseline, demographic and clinical data were recorded, including age, gender, duration of diabetes, HbA1c, fasting blood sugar, post-prandial blood sugar, haemoglobin, and serum albumin levels. Ulcers were graded according to the Wagner classification. Glycaemic control during hospital stay was monitored using periodic blood glucose measurements, and all patients received standardized diabetic care as per institutional protocol. Nutritional status and antibiotic use were recorded, and off-loading measures were uniformly advised.

Before application of dressings, all ulcers were cleaned with normal saline, and surgical debridement was performed when necessary to remove necrotic tissue and slough. In the insulin group, topical insulin dressing was administered using human soluble insulin (Actrapid). A dose of four units (0.1 ml) of insulin diluted in 1 ml of normal saline per 10 cm² of wound surface area was applied directly to the wound bed and covered with sterile insulin-soaked gauze. The dose was not escalated over time, and a maximum limit of 20 units per dressing session was maintained. Insulin was administered using a standard insulin syringe, and the solution was prepared immediately prior to application. In the comparison group, wounds were dressed with sterile gauze soaked in normal saline. Dressings in both groups were performed once daily under aseptic precautions.

All patients received standard diabetic foot care, including optimization of glycaemic control, off-loading of the affected limb, and administration of systemic antibiotics when clinically indicated.

Wound assessment was performed at baseline and subsequently at weekly intervals. The parameters recorded included wound surface area calculated as the product of two maximal perpendicular diameters of the ulcer, percentage of granulation tissue present in the wound bed, and the presence or absence of wound discharge. All wound assessments were performed by trained clinicians to maintain consistency. The clinical outcomes assessed included percentage reduction in wound area, duration of hospital stay, time to complete ulcer closure, and final surgical outcome such as split-thickness skin grafting, secondary suturing, primary epithelialization, or ray amputation.

All data were entered and analysed using IBM SPSS Statistics for Windows, version 26.0 (released 2019, IBM Corp., Armonk, NY). Continuous variables were expressed as mean ± standard deviation, while categorical variables were expressed as number and percentage. Comparisons between the two groups were performed using the independent t-test for continuous variables and the chi-square test for categorical variables. Repeated measures over time were interpreted with caution, and the limitation of not using repeated measures ANOVA or mixed-effects models has been acknowledged. A p-value of less than 0.05 was considered statistically significant.

## Results

Baseline demographic and clinical characteristics were comparable between the two groups. The mean age was similar in the insulin dressing and normal saline groups (57.15 ± 12.71 vs. 56.43 ± 13.04 years). Males constituted the majority of patients in both groups [37 (78.7%) vs. 39 (83.0%)]. There were no statistically significant differences in duration of diabetes, HbA1c levels, fasting or post-prandial blood glucose, haemoglobin, or serum albumin between the groups (p > 0.05). Although a slightly higher proportion of Grade 2 ulcers was observed in the insulin group [21 (44.7%) vs. 12 (25.5%)], the distribution of Wagner grades was comparable overall (Table 1). Although statistically non-significant, the higher proportion of Wagner grade 2 ulcers in the insulin group may have clinical relevance and should be interpreted with caution.

**Table 1:** Baseline and demographic characteristics Baseline and demographic characteristics of patients in insulin dressing and normal saline groups. Data are presented as mean ± standard deviation (SD) for continuous variables and frequency (percentage) for categorical variables. Between-group comparisons were performed using independent t-test for continuous variables and chi-square (χ²) test for categorical variables. A p-value <0.05 was considered statistically significant. Abbreviations: SD: standard deviation; HbA1c: glycated haemoglobin; χ²: chi-square test.

Parameter		Insulin dressing (n = 47)	Normal saline (n = 47)	Test value	p-value
Age (years)	Mean ± SD	57.15 ± 12.71	56.43 ± 13.04	t = 0.27	0.786
Gender	Male	37 (78.7%)	39 (83.0%)	χ² = 0.28	0.600
	Female	10 (21.3%)	8 (17.0%)		
Duration of diabetes (years)	Mean ± SD	11.51 ± 5.04	12.66 ± 5.29	t = −1.08	0.284
HbA1c (%)	Mean ± SD	7.16 ± 0.28	7.16 ± 0.40	t = 0.03	0.974
Fasting blood sugar (mg/dL)	Mean ± SD	120.55 ± 6.34	120.27 ± 6.27	t = 0.22	0.827
Post-prandial blood sugar (mg/dL)	Mean ± SD	161.06 ± 12.75	163.66 ± 12.16	t = −1.01	0.313
Hemoglobin (g/dL)	Mean ± SD	11.49 ± 1.17	11.46 ± 1.27	t = 0.13	0.899
Serum albumin (g/dL)	Mean ± SD	3.45 ± 0.44	3.51 ± 0.35	t = −0.76	0.453
Wagner grade	Grade 1	26 (55.3%)	35 (74.5%)	χ² = 3.77	0.052
	Grade 2	21 (44.7%)	12 (25.5%)		

Granulation tissue formation improved progressively in both groups; however, significantly greater granulation tissue formation was observed in the insulin dressing group from week 1 onwards (p < 0.001). Baseline wound area was comparable between the groups (21.85 ± 25.39 cm² vs. 19.96 ± 12.92 cm²), and absolute wound area measurements during follow-up did not differ significantly. The relatively large standard deviations observed in wound area measurements likely reflect heterogeneity in baseline ulcer size among participants. Nevertheless, the insulin group demonstrated markedly greater wound reduction compared with the saline group (48.44 ± 5.76% vs. 19.15 ± 4.34%, p < 0.001), indicating faster wound healing with topical insulin dressing (Table 2).

**Table 2:** Granulation tissue formation and wound healing parameters Comparison of granulation tissue formation, wound area, and percentage wound reduction between the insulin dressing and normal saline groups over time. Data are presented as mean ± standard deviation (SD). Intergroup comparisons were performed using an independent t-test. A p-value <0.05 was considered statistically significant. Large standard deviations in wound area reflect variability in baseline ulcer size among participants. Abbreviations: SD: standard deviation; cm²: square centimetres

Parameter		Insulin dressing (n = 47)	Normal saline (n = 47)	Test value	p-value
Granulation tissue (%)	Baseline	19.70 ± 5.14	19.98 ± 6.43	t = −0.23	0.818
	Week 1	54.17 ± 5.57	36.34 ± 3.71	t = 18.30	<0.001
	Week 2	77.89 ± 4.17	52.53 ± 4.74	t = 27.30	<0.001
	Week 3	90.40 ± 3.65	66.74 ± 4.06	t = 29.70	<0.001
	Week 4	90.55 ± 3.26	84.94 ± 10.16	t = 3.58	<0.001
Wound area (cm²)	Baseline	21.85 ± 25.39	19.96 ± 12.92	t = 0.45	0.650
	Week 1	16.79 ± 24.88	14.95 ± 6.09	t = 0.49	0.624
	Week 2	11.02 ± 23.84	12.24 ± 4.98	t = −0.34	0.732
	Week 3	8.67 ± 23.01	12.55 ± 4.49	t = −1.13	0.259
	Week 4	8.68 ± 22.99	6.80 ± 3.89	t = 0.55	0.582
Wound reduction (%)	Mean ± SD	48.44 ± 5.76	19.15 ± 4.34	t = 27.90	<0.001

The proportion of patients with wound discharge at baseline was similar in both groups (33 (70.2%) vs. 32 (68.1%)). However, discharge resolved significantly earlier in the insulin group, with no patients showing persistent discharge by week 1 compared with 11 (23.4%) patients in the saline group (p < 0.001). A similar pattern was observed at week 2, where persistent discharge remained in seven (14.9%) patients in the saline group but none in the insulin group. By week 3, discharge had resolved in all patients in both groups. Final surgical outcomes differed significantly between groups, with primary epithelialization observed only in the insulin group (4 (8.5%)), whereas ray amputation was required only in the saline group (3 (6.4%)) (Table 3).

**Table 3:** Wound discharge resolution and final surgical outcomes Comparison of wound discharge resolution and final surgical outcomes between the insulin dressing and normal saline groups. Data are presented as frequency (percentage). Categorical variables were analysed using the chi-square (χ²) test. A p-value <0.05 was considered statistically significant. Abbreviations: χ²: chi-square test; STSG: split-thickness skin graft

Parameter		Insulin dressing (n = 47)	Normal saline (n = 47)	Test value	p-value
Wound discharge	Baseline	33 (70.2%)	32 (68.1%)	χ² = 0.05	0.815
	Week 1	0 (0.0%)	11 (23.4%)	χ² = 13.8	<0.001
	Week 2	0 (0.0%)	7 (14.9%)	χ² = 14.2	<0.001
	Week 3	0 (0.0%)	0 (0.0%)	-	-
	Week 4	0 (0.0%)	0 (0.0%)	-	-
Final surgical outcome	STSG	42 (89.4%)	39 (83.0%)	χ² = 9.78	0.021
	Secondary suturing	1 (2.1%)	5 (10.6%)		
	Primary epithelialization	4 (8.5%)	0 (0.0%)		
	Ray amputation	0 (0.0%)	3 (6.4%)		

Patients receiving an insulin dressing had a significantly shorter duration of hospital stay compared with those receiving a normal saline dressing (14.89 ± 1.90 days vs. 19.21 ± 2.17 days, p < 0.001). Similarly, the time required for complete ulcer closure was substantially shorter in the insulin group (33.4 ± 3.7 days vs. 52.8 ± 5.2 days, p < 0.001). These findings suggest a clinically meaningful improvement in healing outcomes associated with topical insulin dressing (Table 4). 

**Table 4:** Hospital stay and time to complete ulcer closure Comparison of duration of hospital stay and time to complete ulcer closure between insulin dressing and normal saline groups. Data are presented as mean ± standard deviation (SD). Intergroup comparisons were performed using independent t-test. A p-value <0.05 was considered statistically significant. Abbreviation: SD: standard deviation

Parameter	Insulin dressing (n = 47)	Normal saline (n = 47)	Test value	p-value
Hospital stay (days)	14.89 ± 1.90	19.21 ± 2.17	t = −10.27	<0.001
Time to complete ulcer closure (days)	33.4 ± 3.7	52.8 ± 5.2	t = −20.88	<0.001

No patient in either group experienced hypoglycaemia or systemic adverse effects during the study period, based on routine clinical monitoring of blood glucose levels.

## Discussion

DFUs represent a major complication of diabetes mellitus and are associated with significant morbidity, hospitalization, and risk of amputation [3]. The present study compared topical insulin dressing with conventional normal saline dressing in patients with Wagner grade 1 and 2 DFUs. The baseline characteristics in both groups were comparable, with most patients being middle-aged or elderly males. In the present study, males constituted 37 (78.7%) in the insulin group and 39 (83.0%) in the saline group, and the mean duration of diabetes was similar between groups. These findings are consistent with epidemiological data reported by Zhang et al., who demonstrated that DFU patients are typically older individuals with longer duration of diabetes compared with non-ulcerated diabetics [18]. Although baseline comparability reduces the likelihood of major confounding, residual confounding cannot be entirely excluded due to the non-randomized design. Despite the absence of randomization, comparable baseline HbA1c levels and overall Wagner grade distribution reduce, but do not eliminate, the likelihood of major selection bias.

A major finding of the present study was the significantly faster formation of granulation tissue in patients treated with topical insulin. Although baseline granulation tissue percentages were comparable between groups, the insulin group demonstrated markedly higher granulation tissue formation from week 1 onwards. Similar observations have been reported in other studies. Baid et al. reported significantly higher granulation tissue formation in the insulin group (73.7%) compared with the control group (56.6%) at three weeks [19]. Likewise, Nagaraj et al. observed faster granulation tissue development from the second week in patients receiving topical insulin [20]. These findings support the biological role of insulin in promoting fibroblast proliferation, collagen synthesis, and angiogenesis, which contribute to improved wound bed preparation and accelerated healing. However, the assessment of granulation tissue in the present study was based on clinical estimation, which may introduce observer-related variability.

The present study also demonstrated earlier resolution of wound discharge in the insulin-treated group. At baseline, discharge was present in 33 (70.2%) patients in the insulin group and 32 (68.1%) patients in the saline group; however, by week 1, persistent discharge was observed in only 0 (0.0%) patients in the insulin group compared with 11 (23.4%) patients in the saline group. A similar trend was seen at week 2, where discharge persisted in 7 (14.9%) patients in the saline group but none in the insulin group. Comparable findings have been reported by Khan et al., who observed lower infection rates in the insulin-treated group [12 (9.8%)] compared with controls [25 (21.2%)] [21]. Earlier reduction in wound exudate reflects improved control of local inflammation and bacterial colonization, thereby facilitating faster wound healing. Nevertheless, microbiological parameters were not systematically assessed in this study, which limits definitive conclusions regarding infection control.

Another important observation in the present study was the significantly greater reduction in wound size and faster healing rate with topical insulin. The mean percentage wound reduction in the insulin group was 48.44 ± 5.76% compared with 19.15 ± 4.34% in the saline group. Mishra et al. reported wound reduction of 86.9 ± 18.2 % in the insulin group compared with 67.6 ± 14.5 % in controls [22]. Furthermore, a meta-analysis by Hu et al. involving 731 patients reported significantly shorter healing time in patients treated with topical insulin [23]. These findings collectively suggest that insulin enhances wound healing through stimulation of keratinocyte migration, fibroblast proliferation, collagen deposition, and angiogenesis. However, given the observational nature of the present study, these findings should be interpreted as associative rather than indicative of a causal relationship.

The present study also demonstrated significant clinical benefits in terms of shorter hospital stay and faster complete ulcer closure. The mean duration of hospital stay in the insulin group was 14.89 ± 1.90 days compared with 19.21 ± 2.17 days in the saline group, while the time to complete ulcer closure was 33.4 ± 3.7 days versus 52.8 ± 5.2 days, respectively. Similar reductions in healing duration have been reported by Nagaraj et al. and Mishra et al., who also observed significantly shorter healing times in insulin-treated patients [20,22]. Importantly, the final surgical outcomes in the present study were also favourable, with primary epithelialization occurring in 4 (8.5%) patients in the insulin group and none in the saline group, while ray amputation occurred only in the saline group [3 (6.4%)]. These findings indicate that topical insulin not only accelerates wound healing but may also reduce the risk of major surgical interventions and limb loss. However, the observed imbalance in Wagner grade distribution, although not statistically significant, may have influenced outcomes and should be interpreted cautiously. Overall, the present study supports the growing body of evidence that topical insulin is an effective, safe, and inexpensive adjunct for the management of DFUs.

Strengths and limitations

The present study has several strengths, including its prospective design, standardized wound assessment at regular follow-up intervals, and inclusion of clinically relevant outcomes such as granulation tissue formation, wound size reduction, discharge resolution, hospital stay, and final surgical outcomes. Both groups were comparable at baseline, which allowed meaningful comparison of treatment effects. However, certain limitations should be acknowledged. The study was conducted at a single centre with a relatively modest sample size, which may limit the generalizability of the findings. In addition, the observational design without randomization may introduce the possibility of selection bias. Potential confounders such as variations in glycaemic control, nutritional status, antibiotic regimens, and off-loading compliance during follow-up may not have been fully controlled. The follow-up duration was limited to the early healing period, and long-term outcomes such as ulcer recurrence were not assessed. Furthermore, wound measurement using linear dimensions and subjective assessment of granulation tissue may introduce measurement bias. Therefore, larger multicentre studies with longer follow-up are warranted to further confirm the potential benefits of topical insulin in DFU management.

## Conclusions

The present study suggests that a topical insulin dressing may enhance wound healing in patients with DFUs when compared with a conventional normal saline dressing. Patients receiving topical insulin demonstrated earlier granulation tissue formation, greater reduction in wound size, faster resolution of wound discharge, shorter hospital stay, and quicker ulcer closure. Favourable surgical outcomes were also observed in the insulin dressing group. No episodes of hypoglycaemia or systemic adverse effects were noted during the study period, indicating that topical insulin appeared to be well tolerated. However, given the non-randomized design of the study, these findings should be interpreted as associative rather than causal. These observations suggest that topical insulin dressing may serve as a useful adjunct in the management of DFUs; however, larger randomized controlled trials with longer follow-up are required to further validate these findings.
